# Is Mitochondrial Oxidative Stress the Key Contributor to Diaphragm Atrophy and Dysfunction in Critically Ill Patients?

**DOI:** 10.1155/2020/8672939

**Published:** 2020-04-21

**Authors:** Hongjie Duan, Hailiang Bai

**Affiliations:** ^1^Department of Burns and Plastic Surgery, The Fourth Medical Center of Chinese PLA General Hospital, Beijing 100048, China; ^2^Department of Burns and Traumatic Surgery, Hainan Hospital of Chinese PLA General Hospital, Sanya 572013, China

## Abstract

Diaphragm dysfunction is prevalent in the progress of respiratory dysfunction in various critical illnesses. Respiratory muscle weakness may result in insufficient ventilation, coughing reflection suppression, pulmonary infection, and difficulty in weaning off respirators. All of these further induce respiratory dysfunction and even threaten the patients' survival. The potential mechanisms of diaphragm atrophy and dysfunction include impairment of myofiber protein anabolism, enhancement of myofiber protein degradation, release of inflammatory mediators, imbalance of metabolic hormones, myonuclear apoptosis, autophagy, and oxidative stress. Among these contributors, mitochondrial oxidative stress is strongly implicated to play a key role in the process as it modulates diaphragm protein synthesis and degradation, induces protein oxidation and functional alteration, enhances apoptosis and autophagy, reduces mitochondrial energy supply, and is regulated by inflammatory cytokines via related signaling molecules. This review aims to provide a concise overview of pathological mechanisms of diaphragmatic dysfunction in critically ill patients, with special emphasis on the role and modulating mechanisms of mitochondrial oxidative stress.

## 1. Introduction

Respiratory dysfunction is one of the predominant complications of various critical care patients. The common causes for respiratory dysfunction include acute respiratory distress syndrome (ARDS), inhalation injury, blast injury, sepsis, pneumonia, and ventilator-induced lung injury [1, 2], which impair air exchange and ventilation by inducing bleeding, inflammation, infection, edema, exudates increase, mucosal injury, airway obstruction, and atelectasis. However, respiratory muscle atrophy or weakness is always underdiagnosed or ignored by clinicians. Actually, within the complex of critical illness-related weakness, the dysfunction of respiratory muscles, particularly of the diaphragm, represents a highly relevant and distinct clinical problem in intensive care units [3].

Respiratory muscles, including diaphragm, intercostal muscles, abdominal muscles, and accessory muscles, provide the motive power for external respiration. Among these respiratory muscles, the diaphragm is the major inspiratory muscle, which accounts for 60%–80% inhalation power. Similar to limb muscles, diaphragmatic muscle belongs to skeletal muscle. It contains about 55% of slow twitch and fatigue-resistant myofibers (type I) and 45% of fast twitch myofibers (type II). Its roles can be reflected in both the low-intensity, perpetual cycle of breathing and in more rapid and strenuous settings, such as talking, singing, sneezing, defecation, and in situations of acutely increased ventilation [4]. However, respiratory muscles are different from limb skeletal muscles for their unique role in life sustaining. They rhythmically and interminably contract and relax in the whole life process. Maintaining normal respiratory muscle function is extremely important for critically ill patients because their breathing workload increases significantly. In this concise review, we will discuss diaphragmatic dysfunction and its potential pathological mechanisms in critically ill patients, especially emphasize on the contribution of mitochondrial oxidative stress and its potential modulating mechanisms. Those with unilateral or bilateral diaphragm weakness induced by neurological diseases such as medullary transaction and multiple sclerosis and muscular diseases such as muscular dystrophies and dysthyroidism and connective-tissue diseases are not included in this review.

## 2. Contribution of Respiratory Muscle Dysfunction to Respiratory Dysfunction in Critically Ill Patients

As the respiratory pump, respiratory muscle weakness may result in insufficient ventilation, coughing reflection suppression, pulmonary infection, and difficulty in weaning off respirators [5, 6]. All of these further induce respiratory dysfunction and even respiratory failure. In principal, any reduction in respiratory pump function will increase the propensity of respiratory failure, with the level of respiratory workload required to induce respiratory failure directly relate to the level of pump function [7]. In the other words, the lower the inspiratory pump function, the lower the respiratory workload required to induce respiratory failure. In the presence of high drive to breathe in critically ill patients, the imbalance between increased respiratory workload and reduced inspiratory muscle strength causes respiratory distress and CO_2_ retention [8, 9].

It has been verified by animal experiments and clinical trials that respiratory muscle dysfunction or weakness is prevalent in the progress of respiratory dysfunction in various critical illnesses. In animal experiments, continual controlled mechanical ventilation (CMV) for 12–24 h induced diaphragm muscle atrophy indicated by significant decrease of diaphragmatic muscle mass and myofiber cross section area. In addition, diaphragmatic maximal specific force generation decreased by 35%–48% in CMV rats and the magnitude of mechanical ventilation- (MV-) induced force deficit increased with time on the ventilator [10]. Diaphragm muscle dysfunction, which was reflected by a significant decrease of maximum transdiaphragmatic pressure by 49%–63% in vivo and tetanic force by 44%–86% in vitro, was also observed in CMV rabbits for 1–3 days [11]. Our recent study found that there were diaphragm muscle atrophy [12] and diaphragm dysfunction (unpublished work) in severely burned rats. In clinical trials, it has been observed that approximately 64%–79% critically ill patients exhibit diaphragmatic weakness, which is associated with poor clinical outcomes [6, 13–15]. Moreover, it was reported that there was a significant increase in ICU death for MV patients with diaphragmatic weakness (mortality of 35%–49%) compared with those with normal diaphragm function (mortality of 0%–16%) [5, 6, 13–16]. A higher duration of MV and greater incidence of weaning failure have also been observed in those patients with diaphragm weakness [16, 17]. Similar results also were observed in sepsis, chronic obstructive pulmonary disease, cachexia, diabetes, use of corticosteroid, and other critical conditions [18–24]. All of these results suggest that diaphragm dysfunction is common in critically ill patients and is critical for prognosis of various critical illnesses.

## 3. Mechanisms of Diaphragm Atrophy and Dysfunction

The potential mechanisms of diaphragm atrophy and dysfunction include impairment of myofiber protein anabolism, enhancement of myofiber protein degradation, myonuclear apoptosis, autophagy, release of inflammatory mediators, imbalance of metabolic hormones, and oxidative stress. These factors interact with each other and reciprocally cause the development of diaphragm atrophy and dysfunction in a variety of pathological conditions.

### 3.1. Imbalance of Metabolic Hormone and Excessive Inflammation Are Important Upstream Factors in Skeletal Muscle Atrophy and Dysfunction

**Figure 1:**
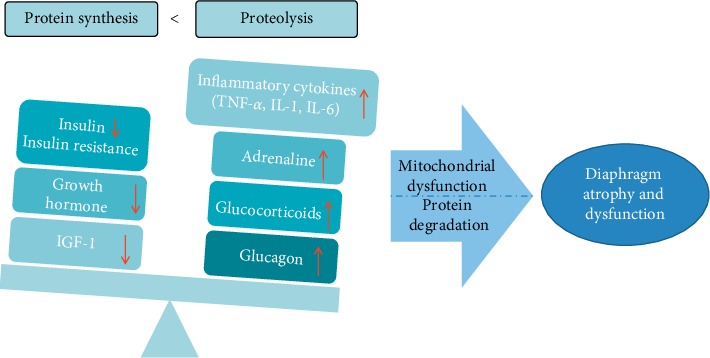
Elevated catabolic hormones, decreased anabolic hormones, and excessive inflammatory factors are up-stream contributors to enhancement of protein degradation and decreased protein synthesis, which further induce diaphragm atrophy and dysfunction in critically ill patients.

Elevated stress hormones in critically ill patients, including adrenaline, glucocorticoids, and glucagon, are important causes in skeletal muscle protein hyper-catabolism. These metabolic changes are exacerbated by insufficient secretion and decreased biological effects of anabolic hormones, including growth hormone, insulin, and insulin-like growth factor-1 (IGF-1). It was reported that dexamethasone injection significantly increased skeletal muscle protein catabolism and chronic low-dose triamcinolone treatment caused a decrease in diaphragm muscle energy status together with a mismatch between glycolysis and oxidative metabolism, which further induced diaphragm wasting and insufficiency of energy supply in rats [25, 26]. In contrast, injection of androgen, an anabolic hormone, significantly attenuated dexamethasone induced skeletal muscle protein degradation through signaling pathways downstream of IGF-1 [25]. In addition, low-dose recombinant human growth hormone treatment significantly improved height, weight, lean body mass and muscle mass, cardiac function, and muscle strength in pediatric burn patients [27]. Elevated inflammatory mediators, such as tumor necrosis factor–*α* (TNF-*α*) and soluble Fas ligand in serum of severely scald rats, were significantly decreased by insulin treatment as indicated by our previous study [28]. These results suggest that increased catabolic hormones and insufficient anabolic hormones induced by various pathological conditions result in decreased diaphragmatic muscle protein anabolism, increased protein degradation, and impairment of energy metabolism, thus leading to muscle atrophy and dysfunction (Figure 1).

Excessive release of inflammatory mediators, such as TNF-*α*, interleukin (IL)-1, and IL-6, activates the ubiquitin-proteasome and apoptotic signaling pathways, which lead to enhanced skeletal muscle protein breakdown in a variety of diseases [29, 30]. The protein wasting further results in muscle atrophy and muscle weakness. In patients with severe trauma, sepsis, and burn injury, serum endotoxin may stimulate monocytes/macrophages. Nuclear factor-*κ*B (NF-*κ*B) in the cytoplasm is then activated through signals such as intracellular phosphoinositide and translocated into the nucleus, which trigger the processes of gene transcription and translation, resulting in a large amount of inflammatory factors secretion and forming a network waterfall effect. Endotoxin was found to accelerate the diaphragm dysfunction process in ventilated rabbits and murine endotoxemia model by augmenting diaphragmatic structural damage, lipid accumulation, release of free radicals, muscle proteolysis, mitochondrial dysfunction, and autophagy of the diaphragm via the toll-like receptor 4 (TLR4)/NF-*κ*B pathway, thus resulting in diaphragmatic contractility impairment [31, 32]. With lipopolysaccharides exposure, diaphragm muscle was associated with a transient activation of NF-*κ*B signaling, subsequently increased atrophic gene expression, enhanced proteasome activity, and persistently impaired contractility [33]. In addition, it was reported that IL-6 and TNF-*α* directly caused ventilatory skeletal muscle atrophy and contractile dysfunction [30, 34]. In comparison, treatment with anti-IL-6 receptor antibody attenuated muscular dystrophy via promoting skeletal muscle regeneration [35]. Our previous study revealed that the level of TNF-*α*, IL-1, and IL-6 in serum of severely burned rats was elevated significantly, which implied the potential role of these inflammatory factors in burn-induced diaphragm atrophy and dysfunction [12, 36]. It seems that excessive inflammatory responses induced by various inflammatory factors initiate proteolytic process, thus resulting in diaphragm atrophy.

### 3.2. Multiple Protein Degradation Pathways Are Involved in Myofiber Protein Catabolism of Diaphragm and Other Skeletal Muscles

**Figure 2:**
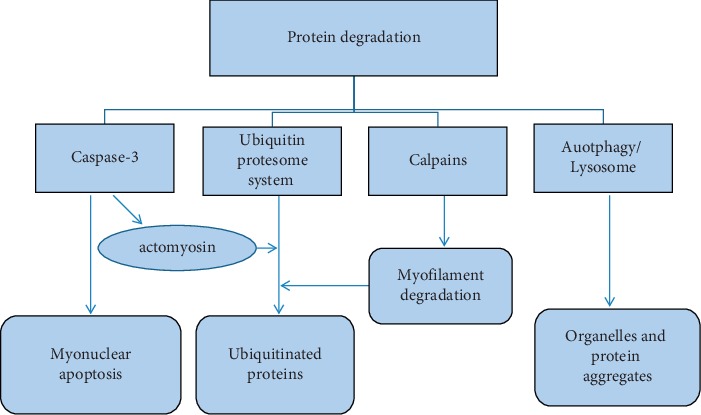
Multiple protein degradation pathways, such as ubiquitin-protesome, caspase-3, calpains, and lysosome, are involved in myofiber protein catabolism of diaphragm and other skeletal muscles.

Myofiber protein degradation is a multistep process involving several proteolytic pathways: lysosomal protease pathway, calcium-dependent protease pathway, ubiquitin-proteasome pathway, and caspase-3 pathway. These proteolytic pathways play different roles, interact with each other, and work together to promote protein breakdown. The ubiquitin-proteasome pathway plays a major role in the diaphragmatic protein degradation in various critical illnesses [37, 38]. However, the ubiquitin-proteasome pathway cannot breakdown actomyosin complexes or myofibrils. It relies on calcium-dependent, calpain-mediated release of myofilaments from the Z-disks or caspase-3-dependent cleavage of actomyosin and producing a characteristic, approximately 14-kD actin fragment and other proteins that are degraded by the ubiquitin-proteasome [28, 39–41]. The underlying proteolytic mechanisms of different myofibers are not completely same. For fast-twitch myofiber atrophy induced by cancer cachexia, sepsis, chronic heart failure, or diabetes, the underlying mechanism is usually related to proteasomal and lysosomal pathways. In contrast, NF-*κ*B activation apparently serves a dual function by inducing both fast-twitch fiber atrophy and slow-twitch fiber degeneration [42]. It was found that not only ubiquitin-proteasome but also caspase-3 and calpains were involved in the process of diaphragmatic atrophy caused by mechanical ventilation and chronic obstructive pulmonary disease [43, 44]. Our previous study revealed that myonuclear apoptosis was induced by death receptor-mediated signaling pathways and other caspase-3 related signals in the diaphragm and tibialis anterior muscles of severely burned rats, which implied that caspase-3 related proteolysis and possible myonuclear loss contributed to the diaphragmatic protein degradation induced by burn injury [12, 28]. Therefore, multiple proteolytic pathways are responsible for protein breakdown of myofibers in the process of diaphragm atrophy and dysfunction (Figure 2).

### 3.3. Mitochondrial Oxidative Stress in the Development of Diaphragm Atrophy and Dysfunction

Mitochondria are commonly considered as one of the main sources of reactive oxygen species (ROS) in skeletal muscle tissues [45, 46]. Mitochondria-generated ROS can be released into cytosol and trigger ROS-induced ROS-release in neighboring mitochondria where the mitochondrial permeability transition pore and the inner membrane anion channel are involved. This mitochondrion-to-mitochondrion ROS-signaling constitutes a positive feedback mechanism for enhanced ROS production leading to excessive oxidative stress [47]. Oxidative stress reflects an imbalance between the systemic manifestation of ROS and a biological system's ability to readily detoxify the reactive intermediates or to repair the resulting damage. Disturbances in the normal redox state of cells can cause toxic effects through the production of peroxides and free radicals that damage all components of the cells, including proteins, lipids, and DNA. There are several potential mechanisms that mitochondrial oxidative stress contributes to diaphragm atrophy and dysfunction as indicated by the previous studies. Firstly, mitochondrial oxidative stress activates the intrinsic apoptotic pathway, proteasome pathway, autophagy, and other protein degradation signaling pathways, resulting in enhanced degradation of diaphragmatic muscle protein [48]. In cultured muscle cells, it was shown that H_2_O_2_-induced mitochondrial oxidative stress activated apoptotic, proteasomal, and autophagic catabolic pathways via increased Bim/Bcl2l11, MAFbx/Atrogin-1, MuRF1, and LC3, which were thought to underlie ventilation-induced diaphragm dysfunction (VIDD) [49]. Secondly, mitochondrial oxidative stress generates free radicals which affect posttranslational modification of muscle proteins and alter their structure and function, including the sarcoplasmic reticulum Ca^2+^ release channel/ryanodine receptor [50, 51], cross-bridge kinetics, and/or reduction in the calcium sensitivity of myofilaments [52, 53]. Moreover, oxidative modification of myofibrillar proteins may enhance their susceptibility to proteolytic processing including 20S proteasome, calpain I, calpain II, and caspase-3 during disuse muscle atrophy [54]. Thirdly, mitochondrial oxidative stress induces a significant reduction of mitochondrial oxidative phosphorylation and increase of glycolysis, which may lead to an overall reduced energy supply to the muscle [43, 55]. Lipid accumulation served as a stimuli to VIDD may result from both the accelerated glycolysis and the decreased breakdown of fatty acids due to compromised mitochondrial function, but the relationship between lipid accumulation and mitochondrial oxidative stress remains unclear [56, 57]. Fourthly, mitochondrial oxidative stress modulates activity of enzymes for neurotransmitters in muscle. It might be responsible for decreased acetylcholinesterase activity in the diaphragm of rats with sepsis [58]. Fifthly, higher ROS levels inhibit IGF-1 signaling cascades. Evidences implicated ROS as downregulators of IGF-1 signaling and inducers of insulin resistance and its pathological sequelae [59]. IGF-1 and insulin are known to promote muscle welfare inducing muscle hypertrophy and stimulate muscle cell proliferation, differentiation, and survival. Finally, mitochondrial oxidative stress impairs differentiation of myoblasts and myotubes. Oxidative stress is known to play a concausal and detrimental role in a variety of multifactorial muscular pathologies characterized by proliferation/differentiation imbalance such as sarcopenia and cachexia [60]. According to their hormetic nature, ROS in muscles may trigger different signaling pathways leading to diverging responses, from adaptation to cell death. Whether a “positive” or “negative” response will prevail depends on many variables, such as among others, the site of ROS production, the concentration of ROS, and the persistence of ROS flow or target cells' antioxidant status [48]. Therefore, it is possible that transiently increased, moderate levels of oxidative stress may represent a potentially protective process, whereas uncontrolled persistence and/or high concentration of ROS may result in diaphragm atrophy and dysfunction.

On the other hand, skeletal muscles with reduced antioxidants adaptation are more prone to oxidative damage. The antioxidants network consists of enzymes, such as catalase, glutathione peroxidase (GPx), thioredoxin reductases, superoxide dismutase (SOD), and soluble antioxidants such as glutathione and vitamin E [48]. It was found that GPx and SOD levels were significantly lower in the diaphragm of severe acute pancreatitis with intra-abdominal hypertension in a rat model [61]. Protein and mRNA expression levels of CuZnSOD, MnSOD, and GPx were found to be decreased or unaltered in the aged muscle even if its activity was increased [62]. Our recent work demonstrated that protein expression of catalase, GPx1, and SOD2 did not alter or even decrease within one week in the diaphragm of severely burned rats, and only catalase and GPx1 had a compensatory increment at 1 or 2 weeks after burn injury [63]. Moreover, increased diaphragmatic levels of SOD2 are essential to achieve the full benefit of exercise-induced protection against VIDD [64]. Theoretically, oxidative stress will result in protective responses, i.e., increased expression of antioxidants. Therefore, not-altered or even decreased antioxidants expression under the condition of oxidative stress may deteriorate this pathological process.

The effect of mitochondrial oxidative stress on diaphragm atrophy and dysfunction was further confirmed by antioxidant treatment. Treatment of VIDD rats with antioxidants such as Trolox, N-acetylcysteine, or the mitochondria-targeted antioxidant peptide SS31 not only averted the activation of proteases such as caspase-3 and calpain and maintained diaphragm protein synthesis but also rescued the MV-induced diaphragmatic myofiber atrophy and contractile dysfunction [65–67]. The successful protection of mitochondria-targeted antioxidants against VIDD also indicates that mitochondria are one of the primary sources of ROS production in the diaphragm during prolonged mechanical ventilation [67, 68]. In addition, a randomized, prospective, placebo-controlled double-blind clinical trial revealed that enteral administration of antioxidants for critically ill adults significantly decreased the duration of mechanical ventilation but not all-cause mortality or the length of stay in the intensive care unit or hospital [69]. Similar results also were achieved in other pathological conditions. Formoterol treatment attenuated the rise in oxidative stress, inflammatory cell infiltration, and the loss of myosin content seen in respiratory and limb muscles of cancer cachectic rats, whereas no effects were observed in the mitochondrial respiratory chain complex activities [70]. Supplementation with tempol or vitamin E recovered diaphragm functional capacity and citrate synthase and lactate dehydrogenase enzymatic activities and reduced the muscle fiber damage and inflammation process in dystrophin-deficient mdx mice [71, 72]. N-acetyl cysteine intervention was also found to be highly effective in preventing hypoxia-induced respiratory muscle weakness and fatigue [73]. These results further confirm the critical role of mitochondrial oxidative stress in the process of diaphragm atrophy and dysfunction, which may be considered a potential therapeutic target.

Taken together, all of these animal and clinical experiments revealed that mitochondrial oxidative stress modulated diaphragmatic protein synthesis and degradation, induced protein oxidation and functional alteration, enhanced apoptosis and autophagy, and reduced mitochondrial oxidative phosphorylation and energy supply and was regulated by inflammatory cytokines. Therefore, it is related to most mechanisms of diaphragm atrophy and dysfunction.

## 4. Signaling Pathways Modulating Mitochondrial Oxidative Stress in the Diaphragm

It has been reported that janus kinases/signal transducer and activator of transcription 3 (JAK/STAT3) signaling, Smad signaling, and Forkhead box protein subclass O (FoxO) family members are closely relevant to mitochondrial oxidative stress in the diaphragm.

### 4.1. Activation of JAK/STAT3 Signaling Is Important in Modulating Mitochondrial Dysfunction and Oxidative Stress in the Diaphragm

The transcription factor STAT3 is activated in response to various cytokines, growth factors, and hormones. After these ligands bind to the transmembrane receptor, STAT3 is activated by JAK-mediated phosphorylation of tyrosine 705 (Y705) and dimerized through reciprocal Src homology 2–phosphotyrosine interaction. The dimeric STAT3 then translocates to the nucleus where it acts as transcription activators and mediates the expression of a variety of genes in response to cell stimuli [74]. It was reported by Tang et al. [42, 43, 75, 76] that the JAK/STAT3 signaling pathway was activated in the diaphragm of critical patients and animals with MV as indicated by up-expression of phosphorylated JAKs and STAT3. Activated STAT3 compromised mitochondrial function and induced oxidative stress. Conversely, oxidative stress also activated JAK/STAT3 signaling in vivo. Inhibition of JAK/STAT3 signaling restored diaphragm muscle contractile dysfunction, prevented oxidative stress-induced protein oxidation and polyubiquitination, and recovered mitochondrial function in rat MV model and cultured muscle cells. Similarly, the JAK/STAT3 signaling pathway was also found to be activated in the diaphragm of experimental cancer cachexia and sepsis, which further induced oxidative stress and diaphragm atrophy and dysfunction [77]. These results reveal the significant contribution of JAK/STAT3 signaling to mitochondrial oxidative stress and muscle atrophy of the diaphragm.

The molecular mechanisms of STAT3 inducing mitochondrial dysfunction and oxidative stress include modulating mitochondrial energy metabolism and activating mitochondrial or atrophic genes transcription. It has been reported that overexpression of STAT3C reduces mitochondrial membrane potential and the efficiency of energy production by uncoupling oxidative phosphorylation from ATP synthesis in cultured cells [75, 78]. Nuclear translocation of phosphorylated STAT3 Y705 upregulated the expression of genes such as Bim, uncoupling protein, and Cox5a, which reduced mitochondrial membrane potential and the efficiency of ATP generation [43]. It seems that in this way phosphorylated STAT3 Y705 impairs mitochondrial energy supply and induces mitochondrial oxidative stress. Also, it was shown that activation of STAT3 directly led to increased expression of myostatin, caspase-3, atrogin, and MuRF, which further resulted in protein ubiquitination and degradation [75–78].

In addition, there is a second phosphorylation site for STAT3 at serine 727 (S727). Activated STAT3 S727 may affect mitochondrial function and suppress oxidative stress by translocating to mitochondria and interacting with components of mitochondrial respiratory chain. Gene associated with retinoid interferon induced cell mortality 19 (GRIM-19), a complex I subunit, acts as a chaperone to recruit STAT3 into mitochondria and enhance the integration of STAT3 into complex I [79]. Mitochondria-localized STAT3 largely exerts its effects through direct or indirect regulation of the activity of the electron transport chain [80]. It was found that mitochondria-localized STAT3 might reduce mitochondrial ROS production by binding to cyclophilin D, suppress autophagy induced by oxidative stress, prevent apoptosis signal-regulating kinase 1 (ASK1)/p38 mitogen-activated protein kinase- (MAPK-) mediated apoptosis, and effectively preserve mitochondria from being degraded by mitophagy [79–82]. It looks like that translocation of mitochondrial STAT3 S727 is a compensatory response to oxidative stress. However, the cell lines used in these studies were fibroblasts, cancer cells, and human embryonic kidney cells. Obviously, they are different from diaphragmatic muscle cells. Therefore, the detailed relationship between mitochondrial STAT3 S727 and oxidative stress in the diaphragm still needs further investigation.

### 4.2. Smad Signaling Is Another Important Modulator of Mitochondrial Oxidative Stress and Muscle Atrophy of the Diaphragm

Smad3 is a transcription factor in the transforming growth factor—*β* (TGF-*β*) signaling pathway. Translocation of phosphorylated Smad3 from the cytosol to nuclei, induction of mitochondrial nicotinamide adenine dinucleotide phosphate hydrogen (NADPH) oxidase 4 (NOX4), and consequent excessive production of cellular ROS as well as activation of mitochondrial apoptotic signaling pathway were detected in a puromycin aminonucleoside-induced podocyte injury model [83]. When activated by myostatin, Smad3, a member of the TGF super family, was found to be sufficient to stimulate atrogin-1 promoter activity, inhibit Akt/mTOR signaling and protein synthesis, and induce tibialis anterior muscle fiber atrophy in mice [84]. In cultured cells and VIDD rats, overexpressed Smad3 induced oxidative stress and protein degradation, whereas inhibition of Smad3 activity not only suppressed oxidative stress and protein degradation but also prevented the reduction in contractility of the diaphragm [85]. Genetic reduction of Smad4 was also found to suppress key aspects of the muscular dystrophy phenotype, including heart and skeletal muscle dysfunction [86]. But it is not sure if Smad4 is an activator of mitochondrial oxidative stress in the diaphragm.

### 4.3. Other Signals Are Involved in the Regulation of Mitochondrial Oxidative Stress and Diaphragm Atrophy

In addition to the STAT3 and Smad3 signaling, FoxO family members, MAPK cascades, NF-*κ*B activator, and calcium-dependent phospholipase A2 (cPLA2) are involved in the catabolic process and oxidative stress of diaphragm atrophy. FoxO family members, such as FoxO1, 3, and 4, directly activate atrophic genes (MAFbx/Atrogin-1 and MuRF1) and autophagy marker gene LC3, promote apoptotic signaling, and regulate mitochondrial function and glucose metabolism [43]. Inhibition of FoxO-specific transcription prevented VIDD by attenuating the activation of the ubiquitin-proteasome system, the autophagy/lysosomal system, and caspase-3 [87]. Free radical scavengers modulate the phosphorylation/activity of some component of the MAPK cascades and beta-dystroglycan and decrease proinflammatory protein NF-*κ*B activator in the diaphragm of dystrophin-deficient mdx mice, implicating the role of oxidative stress in diaphragmatic atrophy via the interaction between MAPK cascade and beta-dystroglycan and NF-*κ*B-mediated inflammation process [88]. cPLA2 also was found to increase mitochondrial superoxide generation and reduce myo-force in the diaphragm of septic mice [89]. However, the exact relationship between oxidative stress and these factors is far away from clarification.

**Figure 3:**
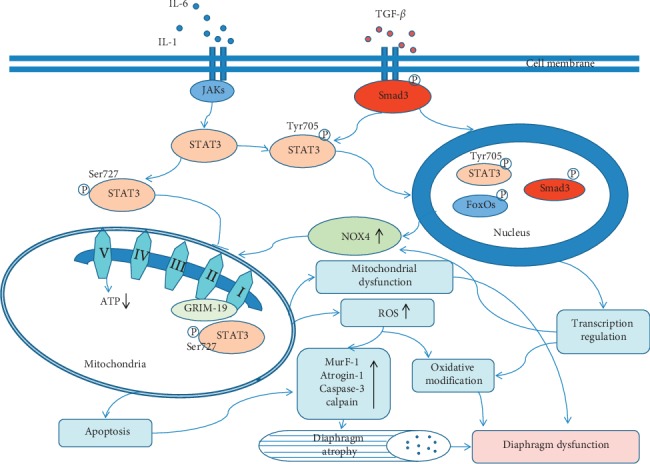
The potential molecular mechanisms of modulating signaling involved in mitochondrial oxidative stress and diaphragm atrophy and dysfunction. ROS: reactive oxygen species; NOX4: nicotinamide adenine dinucleotide phosphate hydrogen (NADPH) oxidase 4.

From the existing evidences, it seems that STAT3, Smad3, FoxOs signaling, and other related signals constitute a signaling network, which induces mitochondrial dysfunction and oxidative stress or directly results in diaphragm atrophy and dysfunction. These signals in the diaphragm interact and cooperate with each other in various pathological conditions. For example, both STAT3 and Smad3 regulate the expression of NOX4 gene, which leads to mitochondrial oxidative stress and protein degradation [43]. Inhibition of Smad3 signaling suppresses STAT3 signaling both in vitro and in vivo [83]. Smad3 regulates transcription of MuRF-1 by increasing FoxO3 binding at a conserved FRE-SBE motif within the proximal promoter region and by increasing FoxO3 protein content and transcriptional activity [90]. In addition, STAT3 and mitochondrial oxidative stress can be activated by each other, which further accelerate the pathological process of diaphragm atrophy and dysfunction. These factors mutually create an environment to prompt mitochondrial oxidative stress and enhance protein degradation, thereby resulting in diaphragm atrophy and dysfunction (Figure 3).

## 5. Summary

Findings in the last decade greatly enhance our knowledge of diaphragm atrophy and dysfunction in the critically ill patient. Indeed, increasing evidences support the critical and central role of mitochondrial oxidative stress in the diaphragm atrophy and dysfunction of critical ill patients. Taken together, the causative results obtained from both preclinical and clinical experiments investigating diaphragm dysfunction point toward a mitochondrial pro-oxidant state (counterbalanced by antioxidants) and regulated by STAT3, Smads, and other related signals. In addition, mitochondrial oxidative stress enhances protein degradation, decreases protein synthesis, and impairs mitochondrial energy metabolism as well as muscular function, thereby culminating in diaphragm atrophy and dysfunction. While the modulating mechanisms are not fully clarified, mitochondrial oxidative stress should be considered a potential therapeutic target for diaphragm atrophy and dysfunction in the critically ill.
